# Swept under the carpet: a qualitative study of patient perspectives on Long COVID, treatments, services, and mental health

**DOI:** 10.1186/s12913-023-10091-9

**Published:** 2023-10-11

**Authors:** Lisa D. Hawke, Anh T. P. Nguyen, Natasha Yasmin Sheikhan, Gillian Strudwick, Susan L. Rossell, Sophie Soklaridis, Stefan Kloiber, Roslyn Shields, Chantal F. Ski, David R. Thompson, David Castle

**Affiliations:** 1 University of Toronto, Toronto, ON Canada; 2 Centre for Addiction and Mental Health, University of Toronto, Toronto, ON Canada; 3 Centre for Mental Health, Swinburne University of Technology, Melbourne, Australia; 4 School of Nursing and Midwifery, Queen’s University Belfast, Belfast, UK; 5 University of Tasmania, Hobart, Australia; 6 Tasmanian Centre for Mental Health Service Innovation, Hobart, Australia

**Keywords:** Long COVID, COVID-19, Mental health, Treatments, Service preferences, Patient-oriented research, Qualitative research, Lived experience

## Abstract

**Background:**

A constellation of often disabling long-term physical symptoms enduring after an acute SARS-COV-2 infection is commonly referred to as Long COVID. Since Long COVID is a new clinical entity, research is required to clarify treatment needs and experiences of individuals affected. This qualitative descriptive study aimed to provide insight into Long COVID treatment and service experiences and preferences of individuals experiencing Long COVID and the intersections with mental health.

**Methods:**

The study was conducted out of a tertiary care mental health hospital, with online recruitment from the community across Canada. A total of 47 individuals (average age = 44.9) participated in one of 11 focus groups between June and December 2022. Five focus groups were conducted with participants who had pre-existing mental health concerns prior to contracting SARS-CoV-2, and six were with people with Long COVID but without pre-existing mental health concerns. A semi-structured interview guide asked about service experiences and service preferences, including mental health and well-being services. Discussions were recorded, transcribed, and analyzed using codebook thematic analysis.

**Results:**

When accessing services for Long COVID, patients experienced: (1) systemic barriers to accessing care, and (2) challenges navigating the unknowns of Long COVID, leading to (3) negative impacts on patient emotional well-being and recovery. Participants called for improvements in Long COVID care, with a focus on: (1) developing Long COVID-specific knowledge and services, (2) enhancing support for financial well-being, daily living, and building a Long COVID community, and (3) improving awareness and the public representation of Long COVID.

**Conclusions:**

Substantial treatment barriers generate considerable burden for individuals living with Long COVID. There is a pressing need to improve treatment, social supports, and the social representation of Long COVID to create integrated, accessible, responsive, and ongoing support systems.

## Background

SARS-CoV-2 infection is usually associated with a short-term illness, with recovery within 7–10 days of onset of a mild infection and 3–6 weeks for severe or critical illness [1]. However, some people experience symptoms over an extended period following the acute illness phase [2]. This phenomenon is commonly known as Long COVID, but is also referred to as post-COVID syndrome, and those experiencing Long COVID are sometimes referred to as COVID long haulers [3]. Long COVID shows many areas of symptomatic overlap with other post-viral syndromes and with myalgic encephalomyelitis/chronic fatigue syndrome, although a connection between these conditions has not been established [4, 5].

While the prevalence of Long COVID has yet to be established definitively, a recent U.S. study suggested that approximately 13.9% of adults infected with COVID-19 manifest some degree of Long COVID symptomatology [6]. Meta-analytic data show that the most common symptoms of Long COVID are extreme fatigue (58%), headache (44%), attentional problems (27%), hair loss (25%), and dyspnea (24%) [7]; ‘brain fog’ and neurological impacts are also common [8]. The Long COVID symptom profile aligns with that of post-viral syndromes from past epidemics such as SARS-CoV-1 and Ebola virus [9]. Mental health challenges also arise. For example, severe COVID-19 has been associated with a doubling of the risk of developing a psychiatric disorder [10], while Long COVID is also associated with an increase in mental health concerns, such as generalized anxiety, depression, sleep disturbance, and post-traumatic stress disorder [11, 12].

The National Institute for Health and Care Excellence (NICE) published clinical practice guidelines that recommended a holistic, integrated, and inter-disciplinary model of care for treatments of Long COVID [2]. However, established interventions and service infrastructures for Long COVID are not in place, and patients struggle with limited support. Previous studies have identified many challenges that Long COVID patients face in accessing care, namely the fragmented healthcare system, limited services tailored to Long COVID experiences, and stigmatization from service providers [13, 14]. Combined with the complexity of Long COVID, a confluence of treatment-related factors places unique burdens upon patients, the effects of which have not yet been determined.

A plethora of research has suggested that negative mental health impacts have emerged from the COVID-19 pandemic [15]. Social interaction and physical activity were disrupted during the pandemic, while mental health challenges increased and the responding mental healthcare system underwent transformations and disruptions [16]. People with Long COVID experience the mental health impacts of living with a new chronic condition, in addition to any mental health impacts that may be directly associated with Long COVID; at the same time, they have to navigate a transformed mental health system while managing various stages of public health restrictions and pandemic-related transformations in public and private life [17].

Our team’s systematic review of registered trials on Long COVID treatments identified the research under way to examine treatments for mental health challenges in the context of Long COVID [18]. That review shows that the research is limited and disparate, leaving an important literature gap and need for research on this condition. Notably, for example, the research under way is not considering the needs of individuals with pre-existing mental health concerns and few include lived experience engagement in their protocols. A related scoping review further underlined that the completed research on interventions for mental health challenges in Long COVID is limited, provisional, and varied, with many areas needing improvement [19].

While there is an emerging body of research describing mental health challenges as sequelae to COVID-19 infection, there remains a lack of research on treatment needs and preferences of individuals whose mental health, well-being and quality of life has been impacted by Long COVID. Research is needed to understand the experiences of this vulnerable population who might have unique experiences and healthcare service needs. An in-depth understanding of their experiences of Long COVID and mental health can best be gained through a qualitative approach.

### Objective

This study aimed to improve our understanding of the Long COVID treatment, service experiences and preferences of individuals experiencing mental health and quality of life impacts of Long COVID, including barriers to adequate treatment. People with lived experience of Long COVID were actively involved throughout the research process to enhance study quality and relevance.

## Methods

This qualitative study consisted of focus group discussions, leading to codebook thematic analysis, to understand lived experience perspectives on Long COVID treatment experiences and preferences. Findings are presented from a sample of participants with Long COVID, as part of a larger study examining the perspectives of patients and service providers across disciplines. The study was conducted out of a tertiary care mental health hospital, with online recruitment from the community across Canada. This study was conducted in compliance with the Canadian Institutes of Health Research Strategy for Patient-Oriented Research [20], with people with lived experience of Long COVID advising throughout the research processes. It was underpinned by a pragmatist epistemology, which emphasizes the importance of practical implications in shaping our understanding of knowledge [21]. Results are reported in accordance with the Consolidated criteria for reporting qualitative research (COREQ) [22]. The engagement of people with lived experience is reported following the GRIPP2 guideline for reporting patient and public involvement (see Table 1) [23].

**Table 1:** GRIPP2 reporting checklist for the engagement of people with lived experience in research

Section & topic	Description
1: Aim	The study team engaged with individuals with Long COVID, with or without pre-existing mental health challenges, in order to support the quality and relevance of the study.
2: Methods	A lived experience advisory group of 6 members was engaged throughout the study; these were individuals with Long COVID, with or without pre-existing mental health concerns. They attended a total of 8 meetings with the research lead and research staff between study initiation and the current reporting, in addition to regular email communication. Advisory group members received $30/h in cash compensation for their contributions.
3: Study results	The contributions of the lived experience advisory group included advising on the study procedures, co-editing the semi-structured interview guide, advising on recruitment materials, sharing recruitment materials in their networks, discussing preliminary codes and themes, and supporting reporting and knowledge translation activities. Lived experience engagement discussions provided substantial reflections that enhanced the relevance of the study procedures. While shifting availability was sometimes a challenge, this was navigated for strong engagement, with 4 advisors remaining in the group to the termination of the project.
4: Discussion and conclusions	The contributions of the lived experience advisory group substantially improved the relevance of the study to the experience of Long COVID. The advisory group’s support with the interview guide development and recruitment efforts enhanced the study. Their insights into the data interpretation further enhanced reporting. The contributions of the group were fundamental to the success of the study and its reporting.
5: Reflections and critical perspective	The lived experience advisory group’s contributions were key to the study’s success. While attrition posed a challenge, the recruitment of a large enough group to continue to gain feedback even in the face of attrition was a mitigating strategy. Open and honest communication with the group minimized other challenges with regard to negotiating consensus on proposed changes. The overall experience of engagement was highly positive.

*GRIPP2* Guidance for Reporting Involvement of Patients and the Public reporting guildeline

### Participants

The sample consisted of 47 participants who reported Long COVID symptoms across 11 focus group discussions. To be eligible, potential participants had to be 18 years of age or older, able to speak English, and self-identify as having Long COVID as confirmed in screening based on the World Health Organization definition, i.e., they had to describe ongoing symptoms at least three months after the onset of an acute COVID-19 infection that continued for at least two months and could not be explained by another diagnosis [24]. Approximate quotas were targeted across demographic variables to maximize participant diversity. A total of 67 potential participants were screened for eligibility, of whom 60 were eligible, 52 consented, and 47 attended a focus group. Reasons for exclusion or dropout included no active Long COVID symptoms (*n* = 1), COVID-19 infection within the previous 3 months (*n* = 2), voluntary withdrawal (*n* = 1), no response (*n* = 9), being unable to attend a focus group (*n* = 4) or outside of demographic quota (*n* = 3). Given the orientation of the study team around examining mental health factors associated with Long COVID, we purposefully sampled approximately half who self-reported having mental health challenges prior to contracting COVID-19 to more fully understand this aspect of mental health experiences (*n* = 23). The goal of this sampling was to gain a breadth of information about different mental health experiences in the context of Long COVID.

### Procedure

Participant recruitment was conducted between June and December 2022. Study information was circulated via email among the research team’s networks and institutional research partners, sent to community Long COVID clinics and support organizations, posted on internal and external institutional websites at the Centre for Addiction and Mental Health (CAMH), and advertised on social media. Potential participants contacted the research team via email, text, or phone. After a brief virtual pre-screening interview to establish inclusion criteria, eligible and interested participants were asked to join a virtual meeting in which a research staff reviewed the study consent form, described the purposes of the study, and explained the procedures. After providing their informed consent, participants completed a demographic survey, hosted on REDCap software [25] on a secure server. Participants were then sequentially invited to a 90-minute focus group discussion. They received a $50 e-gift card for completing a focus group or a $25 card for partial attendance. Participants did not have a relationship with the research team prior to participating in the study. The study was approved by the CAMH Research Ethics Board (#030-2022).

### Data collection

The focus groups were conducted between August 2022 and December 2022, ranging from 61 to 89 min (M = 77.4 min, SD = 7.2 min). Six focus groups were conducted with participants without pre-existing mental health challenges, and five with individuals who reported pre-existing mental health challenges. Present in focus groups were two research staff who facilitated the discussion, together with the participants. Clinical support was available upon request, although this was not utilized. The lead facilitator was a research staff member (ATPN), who was supported by either a post-doctoral research fellow or a PhD student co-facilitator (NYS). The discussions were conducted over WebEx videoconferencing system on a secure server and were video recorded. Notes were taken by the lead facilitator to facilitate the conversations with participants. The chat function was available as a secondary means of adding to the discussion; any comments in the chat were verbalized by the facilitators to stimulate discussion and ensure they were captured in the transcripts. Recordings were transcribed verbatim by research staff or a professional transcription service, then verified by a separate research staff member and uploaded into NVivo (Ver 12) [26]. Transcripts were not reviewed by participants for comments or corrections, but representative quotes were taken to the lived experience advisory group for feedback.

The facilitators used a semi-structured interview guide that was developed in collaboration with the full study team, including lived experience partners. The interview guide consisted of 20 questions divided into three sections: (1) experiences of Long COVID, including mental health, coping, and service experiences, (2) service preferences, including a brainstorming of optimal services, (3) equity, diversity, and inclusion factors affecting Long COVID experiences and service preferences. Each section included suggested prompts to stimulate discussion. For participants with pre-existing mental health challenges, additional questions were added to understand the relationship between pre-existing mental health challenges and Long COVID experiences. The interview guide was pilot tested with members of a research team at CAMH.

### Data analyses

Data were coded and analyzed with NVivo by a research team member (ATPN) using a ‘codebook thematic analysis’ approach [27]. It was determined at the initial data familiarization stage that separating the subgroups of participants with and without pre-existing mental health challenges would not lead to differential findings as the discussions were highly consistent; data from these two subgroups were therefore analyzed together. The lead analyst, together with the research lead (LDH), inductively developed a codebook after the completion of data collection, transcription, and familiarization with the data. The codebook was then entered in NVivo 12. Transcripts were coded using the developed codebook and codes were iteratively refined into themes in collaboration with the research lead through semi-weekly meetings. Our team of lived experience partners also reviewed the preliminary codes and tentative themes for further refinement. Trustworthiness was supported by steps such as prolonged engagement with the transcripts, keeping of audit trails, a commitment to reflexivity, holding team discussions with scientists and lived experience partners, ongoing documentation of team discussions, and reporting on methodological decisions [28]. Results are reported with representative quotes to illustrate the themes generated from the data. Quotes from individuals with pre-existing mental health challenges are indicated as ‘pre-MH,’ and those from individuals without pre-existing mental health challenges are indicated with ‘no pre-MH.’

### Research team positionality

The focus group facilitator and lead analyst is a Vietnamese female who immigrated to Canada. She has completed post-secondary education in psychology and currently holds the position of Research Analyst. Even though she does not experience Long COVID, she has a family member who survived a severe COVID-19 infection that resulted in long-term cognitive and emotional complications. Driven by her personal experience with the family member, she hopes to understand the lived experiences of Long COVID and explore appropriate service pathways for the condition. The research lead is a White female Canadian with a background in psychology, and a research focus on lived experience-engaged mental health and substance use research; she has direct experience of COVID-19 infection, but not Long COVID. Both have experiences navigating the Canadian healthcare system for other conditions from their social positions and reflected on their experiences as part of the analytical process.

## Results

Participant characteristics are presented in Table 2. Participants averaged 44.9 years old (SD = 13.0, range 24 to 69). The majority of participants were women (59.6%). The majority were also White (57.4%), from Ontario (53.2%), and employed (59.6%). However, only 4 (8.5%) participants met this multivariate sociodemographic profile, demonstrating intersectionality in the sample. All participants reported currently experiencing Long COVID symptoms, although only 23 (48.9%) reported receiving an official diagnosis.

**Table 2:** Demographic characteristics of study participants

Demographic characteristic (*N* = 47)		n	%
Age	< 35	9	19.1
	35–54	20	42.6
	55+	11	23.4
	Missing	7	14.9
Gender	Man	18	38.3
	Woman	28	59.6
	Transgender or non-binary	1	2.1
Ethnicity	White	27	57.4
	Indigenous	6	12.8
	South Asian	4	8.5
	East/Southeast Asian	2	4.3
	Multiple ethnicities	3	6.4
	Another ethnicity	3	6.4
	Missing	2	4.3
Location	Western/Central Canada	14	29.8
	Ontario	25	53.2
	Quebec	4	8.5
	Eastern Canada	3	6.4
	Missing	1	2.1
Employment status	Employed	28	59.6
	On disability/sick leave	9	19.1
	Unemployed	4	8.5
	Retired	4	8.5
	Other	2	4.3
Time since COVID-19 diagnosis	3–6 months	9	19.1
	7–12 months	15	31.9
	> 12 months	17	36.2
	Missing	6	12.8
Self-rated mental health	Good to excellent	14	29.8
	Fair or poor	33	70.2
Self-rated physical health	Good to excellent	22	46.8
	Fair or poor	25	53.2

We present the themes derived for two research topics: (1) experiences with healthcare services for Long COVID and (2) service and support preferences among patients with Long COVID. Each topic consists of three themes and several subthemes, illustrated in Table 3.

**Table 3:** Topics, themes and subthemes generated through the analytical process

Topics	Themes	Subthemes
Experiences with healthcare services	Systemic barriers to care	Difficulty accessing family doctors
		Long waits
		Equity and social inclusion barriers
	Challenges navigating the unknowns of Long COVID	Knowledge gap about Long COVID
		Inadequate services for Long COVID
		Illness invalidation by providers
	Negative impacts on patient emotional well-being and recovery	Feeling of abandonment as they navigate the system on their own
		A myriad of emotional repercussions
		Self-stigma as a barrier to accessing care
Service and support preferences	Developing Long COVID-specific knowledge and services	Improving the Long COVID knowledge gap
		Building treatments and services for Long COVID
	Enhancing support for financial well-being, daily living, and building a Long COVID community	Financial support
		Support for daily living & coping
		Community building support
	Improving awareness and the public representation of Long COVID	Education and training
		Building an accurate public representation of Long COVID

### Experiences with healthcare services for Long COVID

Participants’ experiences with healthcare services for Long COVID are represented by three themes: (1) systemic barriers to accessing care, (2) challenges navigating the unknowns of Long COVID, and (3) negative impacts on patient emotional well-being and recovery.

#### Systemic barriers to accessing care

Several systemic barriers, which are typical of the healthcare system, were reported during the focus groups; notably difficulties in accessing family physicians, long waits, and equity and social inclusion barriers were reported. Many participants faced struggles accessing primary care as a first point of contact. Not having a family physician with knowledge of their health history created a fundamental challenge for patients in understanding their Long COVID symptoms, as well as in accessing assessments and navigating treatments.

It’s been hard to find a doctor. I currently don’t have a family doctor, and due to the pandemic there’s been an issue with finding doctors who are accepting patients. I’m going to see one now finally after a few months of waiting, and luckily because of a referral from a friend. But in general it does seem like prior to this friend referring me to the doctor, I don’t know where I would have gone. Nearby walk-in clinics shut down. So, I really don’t know where I would even go to have these Long COVID symptoms addressed. (Focus group 1, no pre-MH)

Furthermore, participants reported lengthy delays in obtaining medical care, such as long waits in emergency departments and for Long COVID-specific treatments.

When I got accepted into the Long COVID centre, the wait time was any time from four to nine months—and that was three months ago. I don’t anticipate hearing from them for quite a while. (Focus group 2, no pre-MH)

Participants also discussed other geographical, social, and economic factors that hindered their access to care for Long COVID. Reported barriers included living in rural areas, having more financial burden, not being permanently employed, or being from a minority group.

I feel like because I’m up in Northern Ontario, that there’s not as much, and the hospitals have longer wait times, and there’s not as many doctors. […] So, like, the fact that where I’m at like geographically, there’s not as many resources available out here. So, it’s not as easy to have someone validate how you feel, or tell you like this is actually what’s going on. (Focus group 3, pre-MH)

#### Challenges navigating the unknowns of Long COVID

Participants encountered Long COVID-specific challenges in accessing care as they navigated the unknowns of Long COVID. These included: (1) knowledge gaps about Long COVID, (2) inadequate services for Long COVID, and (3) illness invalidation from service providers who do not understand the condition.

Participants described major knowledge gaps about Long COVID among both patients and service providers. This encompassed a lack of accurate understanding of Long COVID as an emerging syndrome and the lack of a systematic approach to investigating this condition. Specifically, participants noted that service providers were often ill-informed about the clinical manifestation of Long COVID and were thus unable to provide or refer for effective assessment and treatment.

The lack of information for the general practitioners is really stunning, especially at this point after two and a half years of pandemic. I just find it so frustrating that more info has not come to their hands or they have not been able to be given time to study it. (Focus group 2, no pre-MH)

Participants also acknowledged that they, themselves, were unfamiliar with Long COVID and therefore were unsure of how to obtain help for their symptoms. Some opted for online searches, but found the information available about Long COVID disorganized, inconsistent, and untrustworthy.

I’m on Facebook, but it’s kind of all over the map and I’m not sure if a lot of the time that’s totally applicable. I find, sometimes, it’s way too much information and it’s not a regulated source of information. So, you don’t know what’s good, and what’s bad with the information. (Focus group 4, no pre-MH)

Furthermore, it was clear from participants’ descriptions of their experiences that both they and healthcare providers did not understand the multi-systemic nature of the condition. Both groups tended to overlook the interconnection between physical health and mental health, and the impacts of physical health aspects of Long COVID on mental well-being.

The absence of services tailored to Long COVID was highlighted, as participants discussed the lack of appropriate assessments, official diagnosis, and applicable treatments that constituted a navigational burden. Although a few participants suggested that some services were available, these were often underdeveloped or short term, despite long-lasting symptoms.

My Long COVID clinic stopped trying to do rehab because we all have these secondary conditions. We couldn’t get diagnosed or treated. I did my intake, and then they abruptly cut the actual rehab and shifted on to an educational model. I’ve heard people have similar experiences with others. I haven’t had the energy to try again. (Focus group 5, pre-MH)

Some participants detailed their experiences as a perplexing cycle of testing and consultation which involved referrals to multiple providers without receiving conclusive answers. The logistics involved in completing these appointments, such as time and transportation, as well as a lack of clarity about their health status, could deter patients from accessing services.

Especially if you have multiple symptoms, and you have to see all the different specialists—just wrapping your head around how to go about that, and you know getting all the appointments and all the tests on this. It is a daunting task sometimes. It feels like, will we ever succeed or get to the bottom of our symptoms? Will our doctors help us? Or overall will the general medical community ever find a solution? (Focus group 6, no pre-MH)

As the implementation of Long COVID interventions is in its early stages, participants also reported experiencing trial and error with treatments. They received a wide range of recommendations that they considered to be futile or even harmful, which potentially put their health at risk.

I have had depression before that also, with my anxiety. I found that it was getting worse and my doctor was doing nothing but increasing my SSRIs to the point where I ended up in the ER for toxicity. […] She still refuses to acknowledge the fact that it might be long-term COVID. (Focus group 7, pre-MH)

A prominent theme in the data was that service providers did not believe in Long COVID symptoms; many participants recounted skepticism or even dismissal from service providers. The lack of conclusive medical evidence through testing, combined with knowledge gaps about Long COVID, seemed to lead doctors to question the authenticity of the condition and the veracity of the patient’s symptoms. Participants reported being trivialized and dismissed as providers undermined the significance of their symptoms, mislabeled their condition, and misdirected treatments. This could result in further service delays and symptom exacerbation.

We just have rotating nurse practitioners that come into our local health unit. […] And the first one that I saw, she just totally dismissed COVID and Long COVID. Then she just said, ‘Are you sure it’s not just mental health?’ And she just wouldn’t believe that it was Long COVID. She was trying to convince me that I was just depressed. She wanted to offer me some antidepressants and I know the difference. I know what this is and it’s nothing like that. (Focus group 4, no pre-MH)

#### Negative impacts on patient emotional well-being and recovery

The various service access barriers produced adverse impacts on patient emotional well-being and recovery. Notably, participants expressed (1) a feeling of abandonment as they navigated the system on their own, (2) a myriad of emotional repercussions, and (3) self-stigma as a barrier to accessing care.

Encountering a lack of information and resources, participants found themselves neglected by the healthcare system and were forced take on the roles of researcher, navigator, and self-advocate. They described educating themselves and doctors about Long COVID, inquiring broadly for possible interventions, and advocating for themselves during healthcare visits. Participants believed they had to ‘fight the Long COVID battle’ without proper assistance from the healthcare system.

I feel like I am doing the research myself, going back to my doctor and saying ‘Okay, I am starting to feel like a hypochondriac here. This is now what’s going on. Can we look into this? Can we look into that?’ […] It feels like I am the one trying to educate her and saying can we try this or should we look into this. I don’t feel like they are prepared at all, even after this length of time. (Focus group 2, no pre-MH)

Through negative experiences with healthcare, participants described a myriad of emotional repercussions, which include frustration, anxiety, stress, and hopelessness. Frustration would arise from participants’ communication with service providers, to whom they had to repeatedly explain their symptoms and plead for help, without reciprocal validation from providers. Participants also felt frustrated with the lack of conclusive testing results, effective interventions, and definitive answers.

Unfortunately, it just comes down to, they just tell me to get some rest. And take some vitamins and the time. It’s frustrating, because it doesn’t seem to help some days. (Focus group 8, pre-MH)

Anxiety and stress were also common emotional outcomes. Participants were fearful of the future and wondered whether they would ever receive appropriate help. They were overwhelmed by the pressure of having to justify the legitimacy of their illness to gain access to services and assistance.

Yeah, as they say, I have often felt that I am on trial with WCB [workers compensation board]. I understand that a lot of people, if they are on an insurance company—again, you feel like they think you are faking it or something, when that’s not true. But when the traditional medical test say ‘Yup, heart’s good, chest good,’ why do you have burning chest pain for a year and a half? You know, it is unbelievably stressful—really, really stressful. (Focus group 2, no pre-MH)

Ultimately, this led to a sense of hopeless about the possibility of receiving treatments and recovering.

I’m not going to get anything out of this. I’ll be dead long before they fix Long COVID, but hopefully we can…fix it for the next group who get it. (Focus group 9, pre-MH)

After disappointing experiences with services, many participants considered themselves a burden to the healthcare system and an obstacle to their own healing. A sense of guilt emerged, as participants believed they were initiating a new problem for medicine, taking away care from others, and being not “sick enough” to merit services.

I know that our healthcare system is underwhelmed you know, or you know, overwhelmed and underfunded right now. And you know what? I have that guilty feeling is that here we are bringing something else up that they have to deal with. (Focus group 2, no pre-MH)

Participants also questioned the severity of their illness and blamed their pre-existing physical and mental health conditions for the ongoing Long COVID symptoms.

It is very hard to find people who validate that your experiences are actually real. I actually thought I was going crazy the first year. (Focus group 9, pre-MH)

Due to the complicated process of obtaining treatments, disbelief from providers, and self-stigma, many participants were hesitant to discuss their Long COVID symptoms with health professionals or access medical services. Some chose to address the illness using informal approaches, while others tried to ignore the symptoms and simply wait for improvement. Participants admitted that the tendency to avoid medical care for Long COVID further delayed important treatments and potentially resulted in symptom persistence.

And then I find, you get more frustrated with the whole process. Because you are not really getting any help, so then you don’t want to even look for help. (Focus group 10, no pre-MH)

While challenges with services and providers were widely discussed during the focus groups, a few participants conveyed their gratitude for their experiences with understanding healthcare professionals and effective support programs. Patients felt valued when their providers expressed validation of their condition, showed patience with the ongoing learning gap, and suggested incremental rehabilitation for their symptoms.

So, my insurance case worker, she has been wonderful. She gets, she is the one who helps me the most to get me to stick to a bit of a schedule and you know let’s see how much you can do three days a week. Try and focus on like an online course or something, two hours three days a week just so that to see what I am capable of. (Focus group 8, pre-MH)

### Service and support preferences among individuals with Long COVID

The second area of inquiry was participants’ service and support preferences for Long COVID. Three themes were constructed: (1) developing Long COVID-specific knowledge and services, (2) enhancing supports for financial well-being, daily living, and building a Long COVID community, and (3) improving awareness and public representation of Long COVID.

#### Developing Long COVID-specific knowledge and services

Participants called for more Long COVID-specific supports, which they believed requires the development of knowledge, together with services that should be holistic, integrated, multidisciplinary, accessible and well-informed.

Participants described how knowledge about Long COVID should be built upon scientific investigation, which may lead to applicable testing approaches and official diagnoses. To close the learning gap, participants felt that more inquiry into risk factors, clinical manifestations, and possible interventions for Long COVID should be prioritized. As patients sought explanations for their illness, they felt that research is needed to inform physicians of more deliberate assessment strategies and legitimize the Long COVID diagnosis in the absence of alternative causes.

In addition, participants described a need for a single source of accurate and up-to-date information about Long COVID, to keep patients and families well-informed and guide them in accessing healthcare services. Participants suggested that centralized information, potentially in digital format, could alleviate their confusion and anxiety, and potentially allow for knowledge exchange among patients.

It would be helpful to have it, like, in person but also in a digital ways like an app or computer website. It’s all, like, a one-stop shop for like information, coping mechanisms, people experiencing different symptoms. Because it’s so new, not a lot of people really know much about it or what the symptoms are and what people are going through. To have a one-stop shop with all different types of information and resources and all of that. (Focus group 8, pre-MH)

Participants suggested that Long COVID-specific healthcare services be holistic, integrated and multi-disciplinary, while remaining accessible and well-informed. Emphasizing the complexity of Long COVID, participants recommended that future assessments and interventions leverage the diverse expertise of multiple types of providers to offer more coordinated and comprehensive care. In addition to medical professionals such as family physicians, nurse practitioners, and medical specialists, other types of healthcare professionals should also be involved, notably physiotherapists, occupational therapists, and social workers. The integration of personalized, long-term rehabilitation services was also highlighted to help patients regain independent living.

I have been getting quite a few supports that I found helpful. That if I were to create my own model, I would carry them into it. And the ones that have been the most helpful are physiotherapy, occupational therapy, and psychotherapy for all reasons that we’ve already touched on. I think we all have had challenges with our mental health before. (Focus group 5, pre-MH)

For Long COVID, I would like support for both mental and physical symptoms. I would like a multidisciplinary approach with doctors, nurses available, as well as physical therapists, social workers, psychotherapist who can help support Long COVID. (Focus group 10, no pre-MH)

Participants wished for a holistic approach in which physical, mental, and emotional well-being are equally prioritized. They wanted service providers to acknowledge the interconnection between different components of health and understand that neglecting one component can affect others.

The other thing, and I think [Participant 4] said, it is just this whole notion of integrating services. So, there are aspects there are psychosocial or physical or psychological. Or, it’s all of those things, the whole sort of mind-body-spirit connection. If you’re not feeling well in one of those areas, it’s going to have impact on the others. That’s a given, especially for something that’s so overwhelming as COVID. (Focus group 5, pre-MH)

Participants emphasized that mental health support for Long COVID, through psychologists or psychotherapists, should be considered equally important as services for physical and cognitive health.

The reality is we all need counselling of some sort to support us. (Focus group 2, no pre-MH)

In addition, service affordability, accessibility, and availability were highlighted. Participants expressed their need for the immediate implementation of accessible Long COVID-specific healthcare services, with walk-in services and regular follow-up. Participants wanted flexibility, including virtual, in-person, and hybrid delivery. They also wanted culturally sensitive and trauma-informed services that acknowledge systemic and personal barriers to accessing healthcare.

Balance between medical support, holistic support and mental wellness support. And again, it has to be culturally safe and supported (Focus group 10, no pre-MH).

I think it is much easier to do virtually than in-person, just because of the social anxiety. I don’t wanna have to be physically—just the fear of leaving the house to go and grasp what’s that gonna feel like in-person. It’s just easier to pop in an out virtually and not. I don’t know, it helps. I can’t explain why but it does. (Focus group 7, pre-MH)

#### Enhancing support for financial well-being, daily living, and building a Long COVID community

In addition to formal services, participants also reported seeking informal and instrumental supports, including (1) financial support, (2) support for wellness, daily living and coping, and (3) building a Long COVID community.

Participants reported that Long COVID imposed significant financial burdens, impeding the ability to maintain a stable income and incurring substantial healthcare costs. Financial pressure and associated health anxiety elicited concerns among patients about their future and that of their family. As a result, many participants called for increased financial assistance from the government to help them manage daily expenses and access medical services.

That’s a big piece of it, the financial support, because this entire time, I’ve also just been worried about how I’m going to pay my rent, how I’m going to eat and trying to figure out ways to make money, if this is a new normal. How am I going to survive this? (Focus group 5, pre-MH)

Participants also identified a need for assistance with performing the activities of daily living (*e.g.*, cooking, cleaning), obtaining assistive devices (*e.g.*, wheelchair, cane), and maintaining daily well-being. They wanted support in adopting physical exercises tailored to their fatigue and brain fog, acquiring knowledge about nutrition and sleep hygiene, and practicing coping strategies for mental health, like meditation and breathing.

But the biggest thing that I’m finding is there isn’t any support for us chronic fatigue people to be able to help with our daily living tasks. That is so hard. For me, showering is hard. [Participant 3], I think you were saying earlier about cleaning around your place and stuff like that. It is really difficult… (Focus group 9, pre-MH).

I think the holistic piece is really important. I would love to have somebody to work out a wellness plan with me, you know? And hold me accountable to that—that would be something that would be super helpful. With people who understand it and acknowledge long- term COVID and get some of the struggles that come with it. (Focus group 10, no pre-MH)

Participants expressed a desire to connect with other people who are experiencing Long COVID symptoms through workshops, discussions, or support groups. This would give them a forum to share their stories, exchange support and knowledge, and build an understanding community.

For me, I think support groups would be beneficial. And in that way, you can bounce ideas off each other. You can say somebody’s same symptoms as you do and maybe figure out again, get more ideas from them and what they are doing with their symptoms. And just having to be able to move forward with what’s happening. (Focus group 6, no pre-MH)

Group activities like these would allow for an open and safe environment for individuals with Long COVID to normalize their illness, alleviate social isolation, experience empathy from others, and increase their mental resilience.

#### Improving awareness and the public representation of Long COVID

Lastly, participants emphasized that greater awareness of Long COVID is an essential component of recovery, which can be achieved through education, training, and an accurate public representation.

Participants highlighted the need to enhance the understanding of Long COVID through education and training for service providers, patients, families, and employers. To improve service experiences, participants considered it imperative that physicians receive up-to-date training about Long COVID, show compassion and listen actively to their patients, while recognizing the reality of the condition. Participants suggested information sessions for providers and patients to help recognize Long COVID symptoms, understand the relationship between physical and mental health, and guide treatment seeking. Additionally, they felt that education for family members and employers should aim at supporting daily living and providing appropriate work accommodations.

But making sure people like caregivers, that they have the resources and the information and the… Yeah, if they were able to access an app with all the information and resources to help me when I was not able to help myself, then that would be incredible. (Focus group 8, pre-MH)

I thought, why aren’t they teaching the people in the hospitals about COVID and Long COVID so they have some understanding? […] I just feel like there should have been informational sessions at hospitals with some semblances of understanding of what we were going through. (Focus group 2, no pre-MH)

Participants advocated for building an accurate public representation of Long COVID, to reduce stigma and generate public support for patients.

So, it is unbelievably stressful and it is disturbing to me that we are kind of swept under the carpet. I used to get so frustrated when I watched the daily numbers, you know, they put at the bottom of the newscast—how many people have recovered from COVID, how many people had died, no mention about Long COVID. It’s like it doesn’t even exist. So, when it doesn’t exist, and you say you have it, people kind of shake their head a little bit (Focus group 2, no pre-MH).

Participants expressed the wish that the government and the media would inform the public of the existence and prevalence of Long COVID. Raising awareness of the impairing chronicity and indiscriminate nature of Long COVID, in addition to shifting the blame away from patients, would encourage them to accept the condition, access healthcare services, and reach out to supportive communities.

## Discussion

This study examined patient perspectives on services and treatment needs for mental health and recovery in the context of Long COVID. Combining the experiences of patients with and without pre-existing mental health challenges as no group differences were identified, the results revealed three key themes about patient experiences when accessing services for Long COVID: (1) systemic barriers to care, (2) challenges navigating the unknowns of Long COVID, and (3) negative impacts on emotional well-being and recovery. In addition to a paucity of informed providers and other barriers in healthcare, participants struggled with insufficient knowledge and services for Long COVID, as well as experiencing invalidation from service providers. The unknowns of Long COVID causes emotional distress among patients and provokes self-stigma, resulting in further hesitation to access care. Participants called for substantial improvements in Long COVID care and pinpointed three areas of focus for service implementation: (1) developing Long COVID-specific knowledge and services, (2) enhancing support for financial well-being, daily living, and building a long COVID community, and (3) improving awareness and the public representation of Long COVID.

The current literature on lived experiences of Long COVID and healthcare services in European countries has revealed the absence of a well-defined care pathway for Long COVID; reflected in inadequate services and supports from service providers, this has led to considerable emotional turmoil and service mistrust among patients [13, 14, 29, 30]. Furthermore, patients living with Long COVID can be deterred from accessing services for Long COVID due to numerous institutional burdens, such as the provider shortage and bureaucratic hurdles [31]. Consistent with international research, our study identified similar challenges for patients with Long COVID within the Canadian healthcare context, highlighting the inconsistency in service availability and quality in different settings. Participants identified a Long COVID knowledge gap among both professionals and patients as a treatment barrier, along with social determinants of health and systemic healthcare system barriers that make the management of this condition a matter of navigating the unknown and generating further negative emotional impacts.

To address service gaps and access barriers, participants called for an accessible offering of holistic, integrated, multidisciplinary services specific to Long COVID, alongside accurate and accessible informational resources for clinicians and patients. Indeed, Long COVID clinical practice guidelines, which were developed with people with lived experience, recommend integrated and multi-disciplinary care [2]. This is reflected in a scoping review of Long COVID management, which highlighted the need for a multileveled and collaborative healthcare pathway consisting of coordinated support from primary care, specialists, specialized clinics, non-medical professionals, and community networks [32]. This might include treatments and services for both the physical and mental health components of the Long COVID experiences, using evidence-based therapeutic approaches that have been developed ideally for Long COVID, or that can be generalized to Long COVID from conditions with similar presentations. Attention should also be paid to the systemic challenges typical of the healthcare system, which compound Long COVID-specific challenges. Likewise, patient-engaged service quality principles for Long COVID emerging from the United Kingdom [30] emphasize that providers should set clear clinical responsibilities, provide continuity of care, and lessen the navigational burden for patients. Consistent with these recommendations, our findings support the need for integrated care pathways that include assessment, diagnosis, rehabilitation, and mental health supports while upholding the highest level of standards for inclusivity and accessibility for diverse populations. While developing Long COVID-specific treatments and services, the importance of providing supports for daily living should not be overlooked.

Importantly, patients described invalidation and discrimination during encounters with healthcare professionals, reporting a sense of stigma that compounded the negative emotional impacts of Long COVID. In the healthcare context, epistemic injustice refers to the unfair dismissal of patient experiences and undermining patient credibility as an illness informant [33]. Long COVID symptoms such as brain fog, fatigue, and anxiety rely heavily on patient testimony and are therefore often trivialized or discredited during medical consultations [29, 34]. This is resonant with patient experiences of other ‘invisible’ chronic illnesses like fibromyalgia or myalgic encephalomyelitis/chronic fatigue syndrome (ME/CFS) [35]. Previous research has demonstrated various levels of stigmatization against Long COVID, from direct rejection by healthcare professionals to the anticipation of service discrimination among patients [14, 36]. Illness invalidation leaves Long COVID patients struggling with service navigation and symptom management, while eliciting distress and internalized stigma, and ultimately hindering healthcare service utilization [13, 14, 31]. Experiences of stigma may be particularly strong in the Long COVID sphere, given a degree of societal unrest around COVID-19 and Long COVID, including widespread misinformation and lingering COVID denial [36–38]. This reflects the expressed need to improve the representation of Long COVID among the general public and within the healthcare system. Clinician and public education may be particularly important in this context. Service providers are encouraged to adopt a person-centered, empathetic approach, while providing more active listening and validation of patient experiences despite the lack of medical evidence [13, 29, 39]. However, to achieve this goal, the knowledge gap among clinicians must first be addressed to ensure that they believe in and understand the manifestations of this complex condition.

As the COVID-19 pandemic is on a downturn, interest in the virus and its aftermath appear to be on the decline. However, for the people living with Long COVID, continuing impacts require ongoing investigation and treatment. Patient treatment preferences align with some, but not all, of the research currently under way for mental health and Long COVID [18]. It is therefore critical to expand research addressing appropriate and timely treatments for this condition. While Long COVID-specific treatments and services are required, gains made in this area may find applications beyond the COVID-19 pandemic. Integrated care has been called for to address Long COVID and other conditions with similar manifestations [40, 41]. Needs-based self-management interventions have also been recommended [42], which might align with participants’ expressed need for support in managing their condition and everyday activities. There is therefore a need for improved integrated treatment and care models that emphasize sustainability and generalizability. Rapid knowledge synthesis and novel knowledge translation activities will be required to move findings into practice [43, 44]. Lived experience engagement in these efforts should also be prioritized [45].

The results of this study should be interpreted in the context of its strengths and limitations. Firstly, we adopted a patient-oriented research approach and worked closely with lived experience advisors throughout different stages of the project [20], which enhanced the credibility of the findings and the relevance of study to the experiences of individuals with Long COVID. In addition, focus groups as a data collection method fostered a secure and welcoming environment in which participants could share their stories and connect with others. We also applied a group brainstorm approach for eliciting service preferences, which allowed participants to build an applicable intervention model based on their needs and others’ ideas. Lastly, we assembled a large and relatively diverse sample of participants with and without pre-existing mental health conditions, from heterogeneous backgrounds and geographic areas, thus capturing a broad picture of Long COVID experiences.

However, there are a number of limitations to keep in mind. While the sample size was sufficient for qualitative research, due to the range of symptoms and experiences of Long COVID by individuals, it is possible that some patient experiences may have been missed. There was diversity in the sample across a range of characteristics; however, greater diversity in sociodemographic status and mental health experiences might have generated different findings, with a particular emphasis on minority populations such as Indigenous peoples, LGBTQ + individuals, and newcomer/immigrant populations, among others. It is also possible that those most severely affected by Long COVID were not reached. While discussions revealed that there were at least some participants who may have been influenced by service scarcity specific to living in rural areas as opposed to urban areas, we cannot describe this in detail since we did not collect this demographic variable. It is also of note that the COVID-19 pandemic has been a rapidly evolving phenomenon; the experiences of individuals living with Long COVID earlier in the pandemic may differ from those contracting it more recently.

## Conclusions

Our findings show that individuals living with Long COVID are experiencing substantial barriers to treatment and support, including general healthcare system barriers and additional barriers specific to the condition. Negative experiences of treatment and care are generating considerable burden for this population, which complicate an already complex health experience. There is a pressing need to improve treatment, social supports, and the social representation of Long COVID to create integrated, accessible, continuous, and responsive support systems, with potential application to other conditions with similar manifestations. People with Long COVID should be engaged in research and service development initiatives to create patient-centered service pathways.

## Data Availability

Parts of the data generated and analysed during this study is included in this manuscript. The full dataset is not available for public due to privacy and confidentiality reasons. Please contact the corresponding author for any request regarding the study data.

## References

[1] World Health Organization (2020). Report of the WHO-China joint mission on coronavirus disease 2019 (COVID-19).

[2] Venkatesan P (2021). NICE guideline on long COVID. Lancet Resp Med.

[3] Raveendran AV, Jayadevan R, Sashidharan S (2021). Long COVID: an overview. Diabetes Metab Syndr.

[4] Poenaru S, Abdallah SJ, Corrales-Medina V, Cowan J (2021). COVID-19 and post-infectious myalgic encephalomyelitis/chronic fatigue syndrome: a narrative review. Therapeutic Adv Infect Disease.

[5] Owens B (2022). How long covid is shedding light on postviral syndromes. BMJ.

[6] Perlis RH, Santillana M, Ognyanova K, Safarpour A, Lunz Trujillo K, Simonson MD, Green J, Quintana A, Druckman J, Baum MA, Perlis RH, Santillana M, Ognyanova K, Safarpour A, Lunz Trujillo K, Simonson MD, Green J, Quintana A, Druckman J, Baum MA, Lazer D (2022). Prevalence and correlates of long COVID symptoms among US adults. JAMA Netw Open.

[7] Lopez-Leon S, Wegman-Ostrosky T, Perelman C, Sepulveda R, Rebolledo PA, Cuapio A, Villapol S (2021). More than 50 long-term effects of COVID-19: a systematic review and meta-analysis. Available at SSRN 3769978.

[8] Maury A, Lyoubi A, Peiffer-Smadja N, de Broucker T, Meppiel E (2020). Neurological manifestations associated with SARS-CoV-2 and other coronaviruses: a narrative review for clinicians. Rev Neurol.

[9] Islam MF, Cotler J, Jason LA (2020). Post-viral fatigue and COVID-19: lessons from past epidemics. Fatigue.

[10] Rogers JP, Chesney E, Oliver D, Pollak TA, McGuire P, Fusar-Poli P, Zandi MS, Lewis G, David AS (2020). Psychiatric and neuropsychiatric presentations associated with severe coronavirus infections: a systematic review and meta-analysis with comparison to the COVID-19 pandemic. Lancet Psychiatr.

[11] Bourmistrova NW, Solomon T, Braude P, Strawbridge R, Carter B (2022). Long-term effects of COVID-19 on mental health: a systematic review. J Affect Disord.

[12] Houben-Wilke S, Goërtz YMJ, Delbressine JM, Vaes AW, Meys R, Machado FVC, van Herck M, Burtin C, Posthuma R, Franssen FME (2022). The impact of long COVID-19 on mental health: observational 6-month follow-up study. JMIR Ment Health.

[13] Kingstone T, Taylor A, O’Donnell K, Atherton CA, Blane H, Chew-Graham DN (2020). C, A. : Finding the “right” GP: A qualitative study of the experiences of people with long-COVID. BJGP Open.

[14] Samper-Pardo M, Oliván-Blázquez B, Magallón-Botaya R, Méndez-López F, Bartolomé-Moreno C, León-Herrera S (2023). The emotional well-being of long COVID patients in relation to their symptoms, social support and stigmatization in social and health services: a qualitative study. BMC Psychiatry.

[15] Jenkins EK, McAuliffe C, Hirani S, Richardson C, Thomson KC, McGuinness L, Morris J, Kousoulis A, Gadermann A (2021). A portrait of the early and differential mental health impacts of the COVID-19 pandemic in Canada: findings from the first wave of a nationally representative cross-sectional survey. Prev Med.

[16] Mann DM, Chen J, Chunara R, Testa PA, Nov O (2020). COVID-19 transforms health care through telemedicine: evidence from the field. J Am Med Inform Assoc.

[17] Brüssow H, Timmis K (2021). COVID-19: long COVID and its societal consequences. Environ Microbiol.

[18] Hawke LD, Nguyen ATP, Ski CF, Thompson DR, Ma C, Castle D. Interventions for mental health, cognition, and psychological wellbeing in Long COVID: a systematic review of registered trials. Psychol Med. 2022;52:2426–40. 10.1017/S0033291722002203. 10.1017/S0033291722002203 PMC9300978 35768406

[19] Al-Jabr H, Hawke LD, Thompson DR, Clifton A, Castle D, Ski CF. Interventions to support mental health in people with long COVID: a scoping review. BMC Public Health. 2023;23:1186. 10.1186/s12889-023-16079-8. 10.1186/s12889-023-16079-8 PMC10280822 37340400

[20] Canadian Institutes of Health Research (2011). Canada’s strategy for patient-oriented research: improving health outcomes through evidence-informed care.

[21] Allemang B, Sitter K, Dimitropoulos G. Pragmatism as a paradigm for patient-oriented research. Health Expect. 2021;25(1):38–47. 10.1111/hex.13384 PMC8849373 34748689

[22] Tong A, Sainsbury P, Craig J (2007). Consolidated criteria for reporting qualitative research (COREQ): a 32-item checklist for interviews and focus groups. Int J Qual Health Care.

[23] Staniszewska S, Brett J, Simera I, Seers K, Mockford C, Goodlad S, Altman DG, Moher D, Barber R, Denegri S (2017). GRIPP2 reporting checklists: tools to improve reporting of patient and public involvement in research. BMJ.

[24] World Health Organization (2021). A clinical case definition of post COVID-19 condition by a Delphi consensus, 6 October 2021.

[25] Harris PA, Taylor R, Minor BL, Elliott V, Fernandez M, O’Neal L, McLeod L, Delacqua G, Delacqua F, Kirby J (2019). The REDCap consortium: building an international community of software platform partners. J Biomed Inform.

[26] QSR International Pty Ltd (2020). NVivo 12. In.: QSR International.

[27] Fereday J, Muir-Cochrane E (2006). Demonstrating rigor using thematic analysis: a hybrid approach of inductive and deductive coding and theme development. Int J Qual Methods.

[28] Nowell LS, Norris JM, White DE, Moules NJ. Thematic analysis: striving to meet the trustworthiness criteria. Int J Qual Methods. 2017;16(1):1–13.

[29] Ireson J, Taylor A, Richardson E, Greenfield B, Jones G (2022). Exploring invisibility and epistemic injustice in Long Covid—A citizen science qualitative analysis of patient stories from an online Covid community. Health Expect.

[30] Ladds E, Rushforth A, Wieringa S, Taylor S, Rayner C, Husain L, Greenhalgh T (2020). Persistent symptoms after Covid-19: qualitative study of 114 long Covid patients and draft quality principles for services. BMC Health Serv Res.

[31] Baz SA, Fang C, Carpentieri JD, Sheard L (2023). I don’t know what to do or where to go’. Experiences of accessing healthcare support from the perspectives of people living with long covid and healthcare professionals: a qualitative study in Bradford, UK. Health Expect.

[32] Wolf S, Zechmeister-Koss I, Erdös J (2022). Possible long COVID healthcare pathways: a scoping review. BMC Health Serv Res.

[33] Heggen KM, Berg H (2021). Epistemic injustice in the age of evidence-based practice: the case of fibromyalgia. Humanit Soc Sci Commun.

[34] De Altiery V, Alwan N, Callard F, Berger Z (2021). Listening to long COVID: Epistemic injustice and COVID-19 morbidity.

[35] Åsbring P, Närvänen A-L (2002). Women’s experiences of Stigma in relation to chronic fatigue syndrome and Fibromyalgia. Qual Health Res.

[36] Pantelic M, Ziauddeen N, Boyes M, O’Hara ME, Hastie C, Alwan NA (2022). The prevalence of stigma in a UK community survey of people with lived experience of long COVID. The Lancet.

[37] Jaspal R, Nerlich B. Social representations of COVID-19 skeptics: denigration, demonization, and disenfranchisement. Politics Groups Identities. 2022:11(4):750–70.

[38] Balakrishnan V, Ng WZ, Soo MC, Han GJ, Lee CJ (2022). Infodemic and fake news – a comprehensive overview of its global magnitude during the COVID-19 pandemic in 2021: a scoping review. Int J Disaster Risk Reduct.

[39] Brennan A, Broughan J, McCombe G, Brennan J, Collins C, Fawsitt R, Gallagher J, Guerandel A, O’Kelly B, Quinlan D (2022). Enhancing the management of long COVID in general practice: a scoping review. BJGP Open.

[40] Araja D, Krumina A, Berkis U, Nora-Krukle Z, Murovska M. The Advantages of an Integrative Approach in the Primary Healthcare of Post-COVID-19 and ME/CFS Patients [Internet]. COVID-19 Pandemic, Mental Health and Neuroscience - New Scenarios for Understanding and Treatment. IntechOpen; 2023. Available from: 10.5772/intechopen.106013.

[41] Arnold LM, Gebke KB, Choy EHS (2016). Fibromyalgia: management strategies for primary care providers. Int J Clin Pract.

[42] Hossain MM, Das J, Rahman F, Nesa F, Hossain P, Islam AK, Tasnim S, Faizah F, Mazumder H, Purohit N (2023). Living with long COVID: a systematic review and meta-synthesis of qualitative evidence. PLoS ONE.

[43] Hawke LD, Brown EE, Rodak T, Rossell S, Ski CF, Strudwick G, Thompson DR, Wang W, Xu D, Castle D (2022). Protocol for a systematic review of interventions targeting mental health, cognition or psychological well-being among individuals with long COVID. BMJ Open.

[44] Blutinger EJ, Shahid S, Jarou ZJ, Schneider SM, Kang CS, Rosenberg M (2021). Translating COVID-19 knowledge to practice: enhancing emergency medicine using the wisdom of crowds. J Am Coll Emerg Physicians Open.

[45] Bergerum C, Thor J, Josefsson K, Wolmesjö M (2019). How might patient involvement in healthcare quality improvement efforts work—a realist literature review. Health Expect.

